# The Prognostic Value of C-Reactive Protein Serum Levels in Patients with Uterine Leiomyosarcoma

**DOI:** 10.1371/journal.pone.0133838

**Published:** 2015-08-06

**Authors:** Richard Schwameis, Christoph Grimm, Edgar Petru, Camilla Natter, Christine Staudigl, Wolfgang Lamm, Heinz Koelbl, Michael Krainer, Thomas Brodowicz, Alexander Reinthaller, Stephan Polterauer

**Affiliations:** 1 Department of General Gynaecology and Gynaecological Oncology, Gynecologic Cancer Unit, Comprehensive Cancer Center, Medical University of Vienna, Vienna, Austria; 2 Department of Obstetrics and Gynaecology of the Medical University of Graz, Graz, Austria; 3 Department of Gynaecology, Barmherzige Schwestern Hospital Linz, Linz, Austria; 4 Clinical Division of Oncology, Department of Medicine 1, Comprehensive Cancer Cente, Medical University Vienna, Vienna, Austria; 5 Karl Landsteiner Institute for General Gynecology and Experimental Gynecologic Oncology, Vienna, Austria; CHA University, REPUBLIC OF KOREA

## Abstract

**Objective:**

C-reactive protein (CRP) has previously been shown to serve as a prognostic parameter in women with gynecologic malignancies. Due to the lack of valid prognostic markers for uterine leiomyosarcoma (ULMS) this study set out to investigate the value of pre-treatment CRP serum levels as prognostic parameter.

**Methods:**

Data of women with ULMS were extracted from databases of three Austrian centres for gynaecologic oncology. Pre-treatment CRP serum levels were measured and correlated with clinico-pathological parameters. Univariate and multivariable survival analyses were performed.

**Results:**

In total, 53 patients with ULMS were included into the analysis. Mean (SD) CRP serum level was 3.46 mg/dL (3.96). Solely, an association between pre-treatment CRP serum levels and tumor size (*p* = 0.04) but no other clinic-pathologic parameter such as tumor stage (*p* = 0.16), or histological grade (*p* = 0.07), was observed. Univariate and multivariable survival analyses revealed that CRP serum levels (*HR 2*.*7 [1*.*1–7*.*2]*, *p* = 0.037) and tumor stage (*HR 6*.*1 [1*.*9–19*.*5]*, *p* = 0.002) were the only independent prognostic factors for overall survival (OS) in patients with ULMS. Patients with high pre-treatment CRP serum levels showed impaired OS compared to women with low levels (5-year-OS rates: 22.6% and 52.3%, *p* = 0.007).

**Conclusion:**

High pre-treatment CRP serum levels were independently associated with impaired prognosis in women with ULMS and might serve as a prognostic parameter in these patients.

## Introduction

Uterine leiomyosarcoma (ULMS) reflect the most common type of uterine sarcomas [1]. While the incidence of uterine sarcomas is slowly rising [2], ULMS still is a rare uterine malignancy with an incidence of 3–7 per 100,000 [3] and thus has orphan disease status in the national institute of healths’ list of orphan diseases and in orphanet. ULMS is a tumor of aggressive behaviour and associated with a high mortality rate [1].

Especially in rare and aggressive cancer types, such as ULMS, reliable prognostic parameters are of particular interest. Studies investigating clinical prognostic parameters such as tumor stage or histological grade in patients with ULMS have shown inconsistent results. On the one hand a report from Kapp *et al*. showed an independent influence of tumor stage and histologic grade on survival whereas Hoang *et al*. did not describe an influence of tumor grade on patient’s survival. Zivanovic *et al*. showed that FIGO stage does not perform well in terms of predicting overall survival (OS) in patients with ULMS [4–6]. C-reactive protein (CRP) is a readily available and cheap laboratory parameter, that is widely used in clinical routine as the most important acute phase serum protein to monitor infection. Besides being induced in the acute phase of inflammatory response, CRP has been shown to be elevated in patients with a variety of cancer types and an association with prognosis was found. In particular elevated CRP serum levels have been associated with impaired survival in patients suffering from gynaecologic malignancies including cervical, ovarian, and endometrial cancer [7–9]. In addition several studies investigated the clinical significance of CRP serum levels in soft tissue sarcoma [10]. Therefore, this study set out to evaluate whether pre-treatment CRP serum levels might be used as novel prognostic parameter in patients with ULMS.

## Materials and Methods

### Patients

A total of 53 patients suffering from ULMS, treated between 1996 and 2014 at one of the 3 study centres were enrolled in this study. Clinical data were obtained by chart review. Tumor grading was based on the new international Federation of Gynecology and Obstetrics (FIGO) classification system that was developed to comprise different variables including tumor size, extra uterine spread and invasion of abdominal tissues [11]. Primary tumor assessment was performed by magnetic resonance imagining (MRI) and/or computed tomography (CT) and clinical examination. Treatment consisted of surgery including hysterectomy, bilateral salpingo-oophorectomy, pelvic and/or paraaortic lymphadenectomy in presence of intraoperatively enlarged lymph nodes and surgical cytoreduction in women with extrauterine disease. Radiation therapy, adjuvant and/or palliative chemotherapy were applied, if clinically indicated. All patients were included to our follow-up care program. The follow-up program entails clinical examination and—if recurrent disease is suspected—imaging methods. In the program patients are seen every three to four months for the first three years, every six months up to year five and afterwards annually up to ten years. All patients consented to treatment according to institutional guidelines, and all patients had consented to anonymized assessments and analysis of data and outcome of therapy. The ethics committee of Medical University Vienna approved the study protocol before data collection was started (EK No. 1520/2012). All patient records were anonymized and de-identified prior to analysis. A physical examination by a specialist in internal medicine was performed and presence of infection was ruled out before study inclusion. All patients’ data included in this study are presented in S1 Dataset.

### CRP measurements

Blood samples for CRP serum level evaluation were obtained by peripheral venous puncture prior to treatment start. A commercially available immune-tubidimetric test (Olympus, CRP Latex, Olympus Life and Material Science Europe, Hamburg, Germany) was used for CRP serum level measurements. According to the manufacturer the assay covers a range from 0.5 to 17 mg/dL. Further, an intra-assay variability between 1.64 and 3.34% is claimed by the manufacturer. CRP serum levels of ≤0.5 mg/dL are defined as normal.

### Statistical Analysis

Values are given as mean (standard deviation [SD]). Students’ T- tests and one-way Anova tests were applied to compare mean CRP serum levels and clinico-pathological findings. P-values of <0.05 were considered statistically significant. Survival probabilities were calculated by the product limit method of Kaplan and Meier. Differences between groups were tested using the log-rank test. The results were analysed for the endpoint of OS. Survival times of patients, that were still alive at the last follow up visit, were censored with the last follow-up date. Univariate and multivariable Cox regression models for OS were performed, comprising tumor stage (FIGO I vs. II—IV), histological grade (G1-G2 *vs*. G3), patients' age (< 48.6 *vs*. ≥ 48.6 years), tumor size (<5 *vs*. 5–10 *vs*. >10 cm) and median CRP serum levels (< 3.46 *vs*. ≥3.46 mg/dL). Statistical analysis was performed by use of the commercially available statistical software SPSS 22.0 for MAC (SPSS 22.0, IBM Inc., Armonk, NY).

## Results

Patients’ characteristics are given in Table 1. Mean (SD) CRP serum levels in patients with ULMS were 3.46 (3.96) mg/dL. Interestingly, an association between median CRP serum levels and tumor size but not between median CRP and other clinico-pathological parameters was observed (Table 2).

**Table 1:** Characteristics of patients with uterine leiomyoma.

Parameter	N (%) or mean (SD)
**Total number of patients enrolled**	53
**Age at diagnosis (years)**	48.6 (10.0)
**Pre-treatment CRP serum levels (mg/dL)**	3.5 (4.0)
**Tumor stage**	
FIGO IA	10 (18.9%)
FIGO IB	12 (22.6%)
FIGO II	1(1.9%)
FIGO III	4 (7.5%)
FIGO IV	26 (49.1%)
**Tumor size**	
<5cm	10 (18.9%)
5–10cm	16 (30.2%)
>10cm	22 (41.5%)
Not described	5 (9.4%)
**Histological grade**	
Well differentiated (G1)	5 (9.4%)
Moderately differentiated (G2)	6 (11.3%)
Undifferentiated (G3)	32 (60.4%)
Unknown	10 (18.9%)
**Primary metastatic site**	
Lymph nodes	8 (15.1%)
Lung	15 (28.3%)
Liver	5 (9.4%)
Bone	4 (7.5%)
Other	15 (28.3%)
**Time of follow-up (months)**	32 (1.0–168.0)*
**Status at last observation**	
Alive	28 (52.8%)
Dead	25 (47.2%)

SD: standard deviation, FIGO: International Federation of Obstetrics and Gynecology, CRP: c-reactive protein;

*given as median (range).

**Table 2:** Mean pre-treatment C-reactive protein (CRP) serum levels in patients with uterine leiomyosarcoma categorized by clinico-pathologic findings.

Parameter	Mean CRP mg/dL (SD)	p-value
**FIGO Stage**		0.16^a^
I	2.6 (3.5)	
III-IV	4.1 (4.2)	
**Age**		0.62^a^
<48.6 years	3.2 (4.4)	
≥48.6 years	3.7 (3.5)	
**Tumor size**		0.04^b^
<5 cm	1.0 (0.7)	
5–10 cm	3.0 (3.3)	
>10 cm	4.5 (4.4)	
**Histological grade**		0.07^a^
G1-G2	1.6 (1.5)	
G3	4.2 (4.2)	

^a^ p-value was calculated with t-test.

^b^ p-value was calculated with one-way anova.

SD: standard deviation, FIGO: International Federation of Obstetrics and Gynecology, CRP: c-reactive protein.

**Fig 1:**
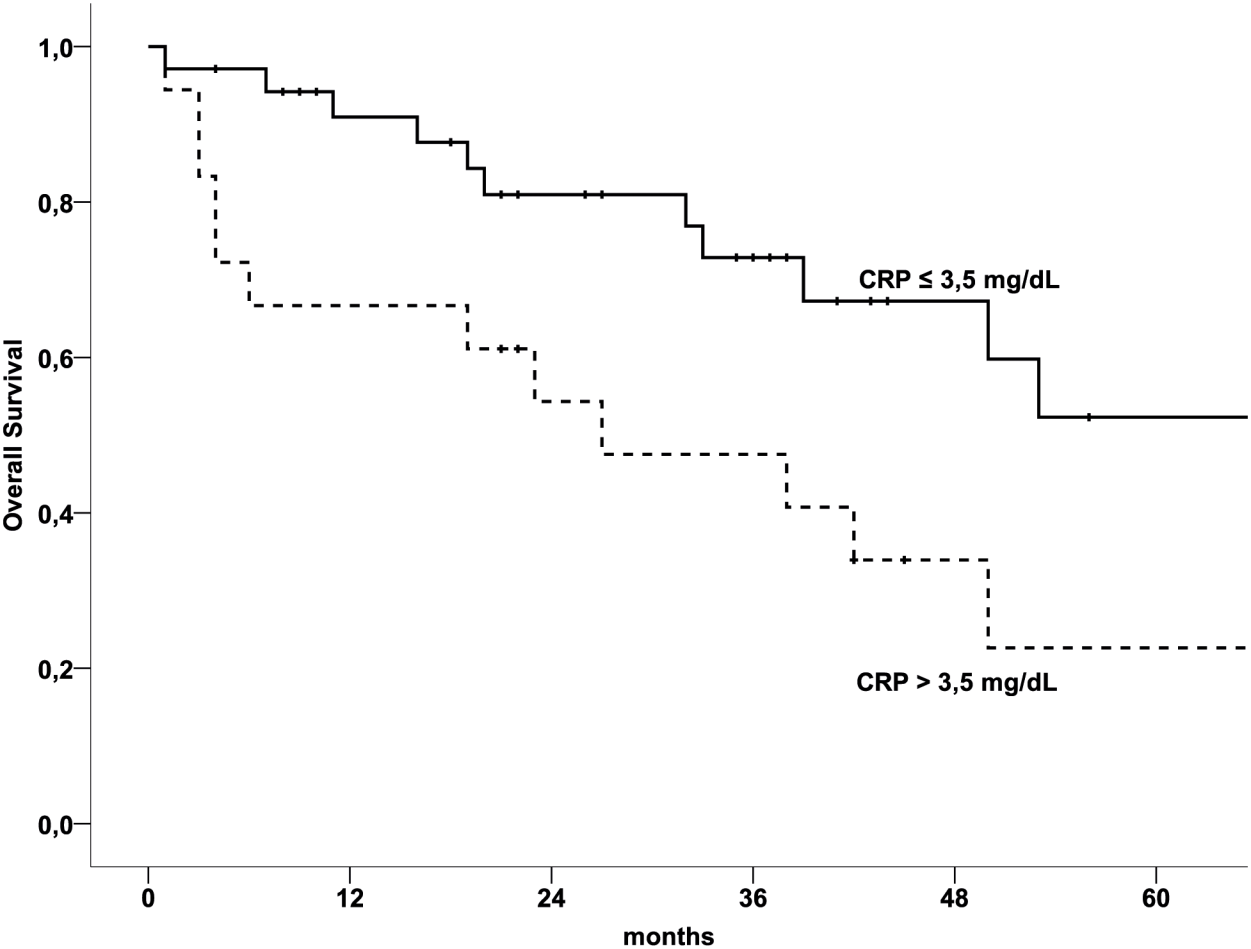
Overall survival in patients with uterine leiomyosarcoma broken down by mean CRP serum levels. CRP: C-reactive protein.

CRP serum levels, tumor stage, patients’ age, tumor size and histological grading were analysed as prognostic parameters for OS by calculating univariate and multivariable survival analysis. Table 3 shows results of the univariate Kaplan-Meier analysis and the multivariable Cox regression model with respect to OS of patients with ULMS. In summary, CRP serum levels, tumor stage and histological grade, but not patients’ age and tumor size were associated with impaired OS in univariate analysis. In multivariable analyses, only CRP serum levels and tumor stage proved to serve as independent prognostic parameters in patients with ULMS. Patients with high pre-treatment CRP serum levels showed impaired OS compared to patients with lower CRP serum levels translating to 5-year OS rates of 22.6% and 52.3% (*p* = 0.007), respectively. A Kaplan-Meier survival curve presenting the association between pre-therapeutic CRP serum levels and OS is shown in Fig 1.

**Table 3:** Univariate and multivariable survival analyses in patients with uterine leiomyosarcoma.

	Univariate		Multivariable	
Parameter	5-year OS rate	p-value	HR (95% CI)	p-value
**CRP**		0.007	2.7 (1.1–7.2)	0.037
≤3.5	52.3%			
>3.5	22.6%			
**FIGO stage**		<0.001	6.1 (1.9–19.5)	0.002
I	72.9%			
II–IV	11.4%			
**Age**		0.83	-	-
<48.6	47.2%			
≥48.6	38.5%			
**Tumor size**		0.5	-	-
<5cm	78.8%			
5–10 cm	54.9%			
>10 cm	32.4%			
**Histological grade**		0.046	2.9 (0.7–12.3)	0.14
G1-G2	60.6%			
G3	22.7%			

CRP: C-reactive protein, OS: overall survival, FIGO: International Federation of Obstetrics and Gynecology, HR: hazard ratio, CI: confidential interval

## Discussion

This is the first report to describe an independent association of pre-treatment CRP serum levels—the most prominent biomarker of inflammation- and prognosis in women diagnosed with ULMS. Compared to low CRP serum levels, high CRP serum levels proved to be an independent prognostic parameter for shorter overall survival translating to significantly lower five-year OS rates in patients with ULMS. This effect was independent of currently clinically used prognostic factors such as FIGO stage and histological grade. Thus, pre-treatment CRP serum levels seem to provide additional prognostic information to established clinico-pathological parameters in patients with ULMS.

Our findings are in accordance with results from previously published studies. CRP serum levels have previously been shown to be useful in predicting outcome of patients with other gynecologic malignancies. A report from Hefler *et al*. showed that high pre-treatment CRP serum levels in patients with ovarian cancer were independently associated with significantly reduced OS [7]. In addition, another report showed that elevated pre-therapeutic CRP serum levels were associated with less favourable prognosis in patients with surgically treated endometrial cancer [9]. However, ULMS originates from smooth muscle cells while other gynaecologic cancers are predominantly of epithelial origin. Hence, ULMS are distinct from other gynaecologic malignancies in terms of response to chemotherapy, tumor biology and survival.

ULMS are considered a subtype soft tissue sarcoma (STS). A report by Szkandera *et al*. showed significantly reduced OS in patients with STS and high pre-therapeutic CRP serum levels [12]. While these results are similar to our results, the data reported here are limited to ULMS. This is of major importance because the group of STS comprises more than 40 heterogeneous tumors with numerous histological subtypes [13, 14]. Throughout distinctive STS subtypes differences in terms of tumor behaviour, chemotherapy response and survival rates have been described [15, 16].

Taken together, the cited reports postulated a wide range of hypothesises regarding the molecular basis of the association between elevated CRP serum levels, inflammation and tumor progression. Inflammation is a critical part of tumor progression and CRP seems to act not only as a biomarker for the process of inflammation and/or tumor progression but also represents a modulator of the immunological system. CRP, as a cytokine, was shown to play an important role in innate host defence and inflammation with various effects on cells and biological processes [17, 18]. At present, the complex molecular basis of the relationship between poor clinical outcome in patients with cancer and elevated CRP serum levels is not fully elucidated, and several possible explanations have been postulated. First, tumor progression may induce tissue inflammation leading to increased CRP serum levels [19]. Second, CRP could represent an indicator of an immune response of the host to tumor antigens or necrosis [20]. Third, the tumor microenvironment consists of cancer cells and inflammatory cells such as granolucytes and lymphocytes. These cells secrete pro-inflammatory cytokines including IL-6, that was shown to induce the production of CRP in hepatocytes. CRP itself seems to induce further inflammatory response and possibly leading to a self-sustaining circle of tumor progression and inflammatory response [17, 19, 21]. Furthermore, Rutkowski et al previously reported, that increased plasma levels of IL-6 occur in approximately 60% of STS patients. Similarly to the results of the current study, increased IL-6 levels—as an indicator for inflammatory response—were associated with poorer survival in patients with STS [22].

In accordance with other trials [4] tumor stage had a strong influence on OS in univariate as well as multivariable analysis in this study. Finally, histologic grading was identified as significant prognostic parameter in univariate but not multivariable analysis of this study. This is in line with other studies regarding histological grade of ULMS [23].

Interestingly, we observed an association of elevated CRP serum levels and tumor size but not with other investigated risk factors for ULMS. Regarding tumor size similar results were reported for patients with STS but not for gynaecologic malignancies [8, 9, 12]. It might be possible that sarcomas with a high tumor load induce higher CRP serum levels, than carcinoma of epithelial origin.

It is of particular interest that CRP serum levels were not associated with patients’ age, since there is evidence that CRP serum levels are elevated in elderly patients compared to younger patients [24]. Interestingly, neither tumor stage nor histologic grading was associated with high CRP levels. The fact, that CRP serum levels were only associated with the tumor size but not with other clinico-pathological parameters underlines the hypothesis, that CRP might provide additional prognostic information and does not only reflect other established prognostic parameters. CRP seems to represent biologically aggressive tumors with impaired prognosis independent of tumor stage and tumor aggressiveness (tumor differentiation) [7].

We think that our findings are interesting from a clinical standpoint and seem biologically plausible. Undoubtedly, the results found in our retrospective study need to be confirmed in future studies. If this was the case, CRP serum levels could be clinically valuable as novel prognostic parameter for ULMS and used for individual risk assessment, patient counselling, and for stratification of patients in future clinical trials. Overall, the clinical consequences of our findings remain to be determined. Whether the inflammatory reaction, which is reflected by increased CRP serum levels, can be treated with anti-inflammatory drugs in order to improve prognosis—as investigated in other solid tumors, such as colorectal cancer—is unclear to date [25].

Several potential limitations of this study have to be recognized when interpreting its results. This is a retrospective study including patients from a relatively long treatment period and different centres. Certainly, the medical care of patients with ULMS has changed within the last years and the influence of this change was beyond the scope of this retrospective trial. Further, the number of patients included to this study is relatively small. On the other hand, ULMS is an orphan disease and this is the first study that evaluated pre-therapeutic CRP serum levels as prognostic factor in this population.

At present, CRP is widely used in clinical routine to monitor acute and chronic inflammation and is a cost-effective and readily available prognostic parameter. Especially in the lack of reliable prognostic serum parameters for women diagnosed with ULMS, CRP serum levels might be clinically useful.

In conclusion we present CRP serum levels as a novel independent prognostic parameter in women diagnosed with ULMS, a rare cancer type vastly distinctive from both epithelial gynaecologic malignancies and other STS. Validation of our results in future trials is warranted.

## Supporting Information

S1 Dataset Data of patients suffering from uterine leiomyosarcoma (ULMS). (XLSX)

## References

[1] D'Angelo E, Prat J. Uterine sarcomas: a review. Gynecol Oncol. 2010;116(1):131–9. 10.1016/j.ygyno.2009.09.023 . 19853898

[2] Ueda SM, Kapp DS, Cheung MK, Shin JY, Osann K, Husain A, et al Trends in demographic and clinical characteristics in women diagnosed with corpus cancer and their potential impact on the increasing number of deaths. Am J Obstet Gynecol. 2008;198(2):218 e1–6. 10.1016/j.ajog.2007.08.075 . 18226630

[3] Brooks SE, Zhan M, Cote T, Baquet CR. Surveillance, epidemiology, and end results analysis of 2677 cases of uterine sarcoma 1989–1999. Gynecol Oncol. 2004;93(1):204–8. 10.1016/j.ygyno.2003.12.029 . 15047237

[4] Kapp DS, Shin JY, Chan JK. Prognostic factors and survival in 1396 patients with uterine leiomyosarcomas: emphasis on impact of lymphadenectomy and oophorectomy. Cancer. 2008;112(4):820–30. 10.1002/cncr.23245 . 18189292

[5] Zivanovic O, Leitao MM, Iasonos A, Jacks LM, Zhou Q, Abu-Rustum NR, et al Stage-specific outcomes of patients with uterine leiomyosarcoma: a comparison of the international Federation of gynecology and obstetrics and american joint committee on cancer staging systems. Journal of clinical oncology: official journal of the American Society of Clinical Oncology. 2009;27(12):2066–72. 10.1200/JCO.2008.19.8366 19255317 PMC3646302

[6] Hoang HL, Ensor K, Rosen G, Leon Pachter H, Raccuia JS. Prognostic factors and survival in patients treated surgically for recurrent metastatic uterine leiomyosarcoma. International journal of surgical oncology. 2014;2014:919323 10.1155/2014/919323 25045534 PMC4090477

[7] Hefler LA, Concin N, Hofstetter G, Marth C, Mustea A, Sehouli J, et al Serum C-reactive protein as independent prognostic variable in patients with ovarian cancer. Clinical cancer research: an official journal of the American Association for Cancer Research. 2008;14(3):710–4. 10.1158/1078-0432.CCR-07-1044 . 18245530

[8] Polterauer S, Grimm C, Tempfer C, Sliutz G, Speiser P, Reinthaller A, et al C-reactive protein is a prognostic parameter in patients with cervical cancer. Gynecol Oncol. 2007;107(1):114–7. 10.1016/j.ygyno.2007.06.001 . 17617445

[9] Schmid M, Schneitter A, Hinterberger S, Seeber J, Reinthaller A, Hefler L. Association of elevated C-reactive protein levels with an impaired prognosis in patients with surgically treated endometrial cancer. Obstetrics and gynecology. 2007;110(6):1231–6. . 18055714 10.1097/01.AOG.0000292085.50987.f2

[10] Nakamura T, Matsumine A, Matsubara T, Asanuma K, Uchida A, Sudo A. Clinical significance of pretreatment serum C-reactive protein level in soft tissue sarcoma. Cancer. 2012;118(4):1055–61. 10.1002/cncr.26353 . 21761398

[11] Prat J. FIGO staging for uterine sarcomas. International journal of gynaecology and obstetrics: the official organ of the International Federation of Gynaecology and Obstetrics. 2009;104(3):177–8. 10.1016/j.ijgo.2008.12.008 . 19135669

[12] Szkandera J, Gerger A, Liegl-Atzwanger B, Absenger G, Stotz M, Samonigg H, et al Validation of the prognostic relevance of plasma C-reactive protein levels in soft-tissue sarcoma patients. British journal of cancer. 2013;109(9):2316–22. 10.1038/bjc.2013.595 24084772 PMC3817333

[13] Dufresne A, Cassier P, Heudel P, Pissaloux D, Wang Q, Blay JY, et al [Molecular biology of sarcoma and therapeutic choices]. Bulletin du cancer. 2015;102(1):6–16. 10.1016/j.bulcan.2014.12.005 . 25609490

[14] Scurr M. Histology-driven chemotherapy in soft tissue sarcomas. Current treatment options in oncology. 2011;12(1):32–45. 10.1007/s11864-011-0140-x . 21359911

[15] Jain S, Xu R, Prieto VG, Lee P. Molecular classification of soft tissue sarcomas and its clinical applications. International journal of clinical and experimental pathology. 2010;3(4):416–28. 20490332 PMC2872748

[16] Samuels BL, Chawla S, Patel S, von Mehren M, Hamm J, Kaiser PE, et al Clinical outcomes and safety with trabectedin therapy in patients with advanced soft tissue sarcomas following failure of prior chemotherapy: results of a worldwide expanded access program study. Annals of oncology: official journal of the European Society for Medical Oncology / ESMO. 2013;24(6):1703–9. 10.1093/annonc/mds659 . 23385197

[17] Groblewska M, Mroczko B, Sosnowska D, Szmitkowski M. Interleukin 6 and C-reactive protein in esophageal cancer. Clinica chimica acta; international journal of clinical chemistry. 2012;413(19–20):1583–90. 10.1016/j.cca.2012.05.009 . 22609487

[18] Volanakis JE. Human C-reactive protein: expression, structure, and function. Molecular immunology. 2001;38(2–3):189–97. . 11532280 10.1016/s0161-5890(01)00042-6

[19] Coussens LM, Werb Z. Inflammation and cancer. Nature. 2002;420(6917):860–7. 10.1038/nature01322 12490959 PMC2803035

[20] Ramsey S. The role of the systemic inflammatory response as a biomarker in immunotherapy for renal cell cancer. Molecular diagnosis & therapy. 2009;13(5):277–81. . 19791832 10.1007/BF03256333

[21] Wigmore SJ, Fearon KC, Sangster K, Maingay JP, Garden OJ, Ross JA. Cytokine regulation of constitutive production of interleukin-8 and -6 by human pancreatic cancer cell lines and serum cytokine concentrations in patients with pancreatic cancer. International journal of oncology. 2002;21(4):881–6. . 12239630 10.3892/ijo.21.4.881

[22] Rutkowski P, Kaminska J, Kowalska M, Ruka W, Steffen J. Cytokine serum levels in soft tissue sarcoma patients: correlations with clinico-pathological features and prognosis. International journal of cancer Journal international du cancer. 2002;100(4):463–71. 10.1002/ijc.10496 . 12115531

[23] Giuntoli RL 2nd, Metzinger DS, DiMarco CS, Cha SS, Sloan JA, Keeney GL, et al Retrospective review of 208 patients with leiomyosarcoma of the uterus: prognostic indicators, surgical management, and adjuvant therapy. Gynecol Oncol. 2003;89(3):460–9. . 12798712 10.1016/s0090-8258(03)00137-9

[24] de Maat MP, Kluft C. Determinants of C-reactive protein concentration in blood. Italian heart journal: official journal of the Italian Federation of Cardiology. 2001;2(3):189–95. . 11305530

[25] Ye XF, Wang J, Shi WT, He J. Relationship between aspirin use after diagnosis of colorectal cancer and patient survival: a meta-analysis of observational studies. British journal of cancer. 2014 10.1038/bjc.2014.481 . 25180765 PMC4260025

